# Venous thromboembolism rates after hip and knee arthroplasty and hip fractures

**DOI:** 10.1186/s12891-020-3100-4

**Published:** 2020-02-12

**Authors:** Viswanath Mula, Sunny Parikh, Sivakolundu Suresh, Alex Bottle, Mark Loeffler, Mahbub Alam

**Affiliations:** 1 Department of Orthopaedics, Colchester General Hospital, Turner Road, Mile End, Colchester, Essex, CO4 5JL UK; 2 Imperial College London, London, UK

**Keywords:** VTE, THA, TKA, Hip fractures, PACS

## Abstract

**Background:**

The ideal thromboprophylaxis regime following lower limb arthroplasty and proximal femur fractures remains controversial. Guidelines disagree on the type of chemical prophylaxis, its dose or duration. This article describes a method of monitoring venous thromboembolism (VTE) rates following Total Hip (THA), Total Knee Arthroplasty (TKA) and surgery for hip fractures (NOF#).

**Methods:**

Over 3 years, all patients investigated for VTE were analysed using Picture Archiving Communications System (PACS). All positive scans were then cross-referenced using PACS and local registry data to see if they had undergone THA, TKA or NOF# in the preceding 90 days. Mortality data were obtained from the national administrative database, Hospital Episode Statistics.

**Results:**

Five thousand seven hundred eighty-eight patients underwent investigation for VTE and there were 29 diagnoses of PE and 24 of DVT. There was a 0.77% rate of symptomatic DVT after THA, 0.05% after TKA and 0.55% after NOF #. The rate of confirmed symptomatic PE for THA was 0.46, 0.27% for TKA and 0.96% for NOF #. Mortality at one-year post-THA was 0.6, 0.6% for TKA and 25.9% after NOF#. All patients contacted either remained within the catchment area for the minimum 90 postoperative days or died within the catchment area.

**Conclusions:**

The 90 day post-operative prevalence of symptomatic VTE of 1.2, 0.3 and 1.5% in THA, TKA and NOF # respectively are similar to other studies using symptomatic and imaging positive VTE as their endpoint. The study uses a method of collecting data which can be utilised in centres where PACS is available.

## Background

The ideal chemo-thromboprophylaxis agent to use and its duration for Total Hip Arthroplasty (THA), Total Knee Arthroplasty (TKA) and surgery for neck of femur fractures (NOF#) remains controversial. The National Institute of Clinical Excellence (NICE) guidelines for THA and NOF#, in England and Wales, recommends mechanical prophylaxis combined with LMWH or Fondaparinox for 4 weeks post-surgery [1]. The American College of Chest Physicians (ACCP) Grade-1A guidelines recommend either LMWH or Fondaparinox or warfarin (target international normalised ratio of 2.5, range 2.0–3.0) for a minimum of 10 days. The guidelines for TKA are identical but also include the use of pneumatic compression devices [2]. The doses of pharmaceutical prophylaxis are not specified. Orthopaedic surgeons remain reluctant to implement the guidelines fully [3–5]. In our department we utilise 20 mg of Enoxaparin, half of the recommended 40 mg daily dose [6], for the duration of the in-patient stay [7]. Comparison of our regime with the recommended 40 mg Enoxaparin dose has suggested no differences in hospital 30-day readmission rates, venous thromboembolism (VTE) rates and postoperative haemorrhage rates in the management of neck of femur fractures [7]. Our aim was to use the Patient Archiving Communication System (PACS) to find patients with symptomatic VTE occurring within 90 days of the index procedure of Total Hip Arthroplasty (THA), Total Knee Arthroplasty (TKA) and surgery for Neck of Femur fractures (NOF#). This would allow the determination of the rate of symptomatic PE and DVT following hip and knee replacements and hip fractures using this regime. Our methodology of data collection could be adopted at other centres to ultimately enable us to gather larger volumes of data and therefore compare different thromboprophylaxis regimes.

## Methods

The study included all the consecutive patients discharged from Colchester General Hospital (CGH) and Ramsey Oaks Private Hospital (ROPH) in Colchester after THR, TKR or surgery for NOF# fractures in a 3 year period between November 2006 and November 2009. No patients with NOF# were treated at ROPH. The hospitals serve a mixed urban and rural population of 370,000 [8]. Prior to discharge, radiographs were used to check the adequacy of the index procedure. All post-operative patients with symptomatic VTE underwent further diagnostic imaging at CGH. The need for ethics approval was waived by Colchester Hospital IRB since data checking was anonymous.

Information on mortality within hospital and within 1 year of surgery was obtained from national administrative data, Hospital Episode Statistics (HES) linked with the Office for National Statistics death certification fields, for Colchester General Hospital only, as HES does not cover private hospitals other than Treatment Centres (ISTCs). The following Office of Population, Censuses and Surveys Classification of Surgical Operations and Procedures (OPCS) codes were used to define the elective procedures: W371, W379, W381, W389, W391, W399 (THR), W401, W409, W411, W419, W421, W429 (TKR). NOF# was defined using a primary diagnosis of S720-S722 in the first consultant episode without a symptom or sign code (ICD10 R chapter), emergencies only. Episodes were linked into spells and transfers linked into super-spells (complete admissions) to avoid multiple counting.

### Thromboprophylaxis

All elective THR and TKR patients received once daily 20 mg of Enoxaparin (Sanofi-aventis, Paris, France) started 12 h prior to the index procedure [7]. The NOF# patients began LMWH prophylaxis on admission. We used graduated compression stockings from admission to up to 6 weeks post index procedure. Peri-operative pneumatic compression was provided using Flowtron® Universal calf pumps (ArjoHuntleigh International Ltd., Beds, UK) for 24 h. Compression stockings and pneumatic compression was not used if contraindicated (Peripheral vascular disease, severe congestive cardiac failure, local skin problems). Early mobilisation with physiotherapy was utilised. Patients were checked for adequate hydration and intravenous fluid therapy was extended where necessary. The LMWH was stopped on discharge. Automatic blood transfusion was undertaken if the postoperative haemoglobin level was ≤8.0 g/dl or the patient was symptomatic secondary to low levels of haemoglobin.

### Diagnosis of VTE

Suspected VTE patients are assessed using a structured clinical assessment which ensures all post-operative patients are treated as high risk. Clinically suspected deep vein thrombosis (DVTs) underwent a Doppler Compression B-mode Ultrasound scan examination of deep, superficial and common femoral veins [9]. During the study period no patients required venography to aid the diagnosis of lower limb DVT. All the patients with suspected pulmonary embolism (PE) underwent either a Computerised Tomographic Pulmonary Angiogram (CTPA) or a Ventilation-Perfusion (V/Q) Scan. Only high probability on the V/Q scan was considered as a positive pulmonary embolism using the criteria based on the Prospective Investigation of Pulmonary Embolism Diagnosis (PIOPED) [10]. Suspected VTE cases were all reviewed at CGH as facilities to diagnose VTE do not exist at ROPH.

**Fig. 1:**
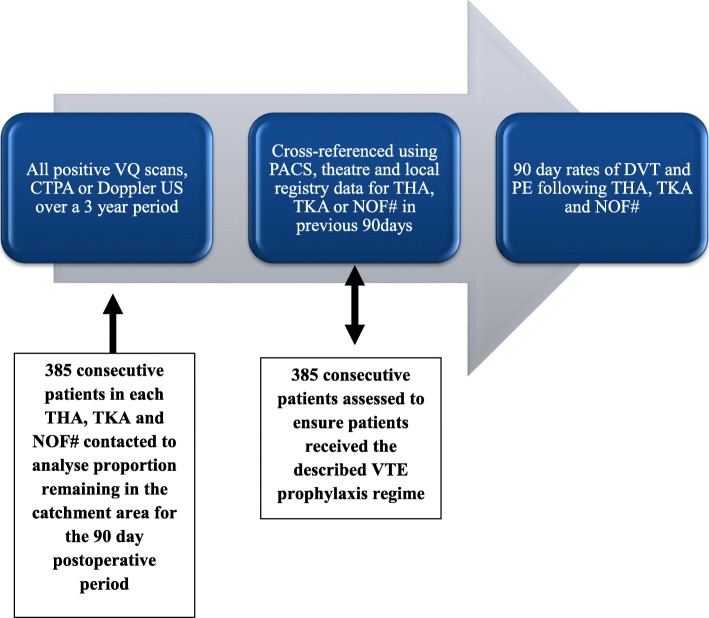
A flow chart explaining the sequence for calculating the rate of postoperative 90 day VTE. The blue boxes show the 3 stages in the collection of data to calculate the VTE rates. The 2 white boxes show how the method was validated

The PACS became online in 2006 with all diagnostic imaging at CGH being stored on the system. A similar system was used at ROPH. PACS was used to identify all recorded positive lower limb Döppler ultrasound scans, Computed Tomographic Pulmonary Angiograms (CTPA) and Ventilation/Perfusion scans (V/Q) within the 3 year study period. The positive results were then checked using PACS at both hospitals to assess if the symptomatic and proven VTE patients had undergone imaging for THA, TKA or surgery for NOF# in the preceding 90 days. This was additionally cross referenced with hospital and operating theatre records, and local arthroplasty registry data (Fig. 1). The demographic data and length of hospital stay were available from the clinical coding information.

### Population movement post-surgery

We assessed the postoperative movement of patients in the 90 days following THA, TKA or surgery for NOF# by contacting 385 consecutive patients from each of the 3 surgical procedure groups or their carers in cases where co-morbidity or social circumstances dictated (e.g. living in nursing or residential home) by telephone. Contacting 385 patients for each for the THA, TKA and NOF# groups would allow a 95% confidence interval, no wider than 0.10 (10%) for each group [11], that patients developing VTE post-surgery would have remained within our catchment area and, therefore, would have attended CGH for further investigation and management.

### Compliance to VTE thromboprophylaxis regime

We checked compliance to the VTE regime described above by assessing 385 consecutive clinical notes each for THA, TKA and NOF # [11]. Patients taking warfarin or other anticoagulant chronically were excluded.

## Results

During the 3 year study period there were 1954 THAs, 1870 TKAs and 1451 procedures for NOF #, giving a total of 5275 patients. The female to male ratio was 1.7:1. The mean ages were 73.4 years for THA, 70 years for TKA and 82.3 years for NOF#.

Two thousand three hundred fifty-six patients underwent either CTPA or V/Q scan for symptomatic PE and 3432 patients underwent Döppler US for symptomatic DVT (total number of scans 5788). After cross-referencing all the positive scans for preceding orthopaedic surgery using PACS and local registry data we found 28 confirmed diagnoses of symptomatic PE and 24 confirmed cases of symptomatic DVT. The female to male ratio was 2.25 to 1.

The mean time to presentation for symptomatic PE was 22.5 days (SD +/− 21), 9 days (SD +/− 21.7) and 22 days (SD +/− 19.8) and for THA, TKA and NOF# respectively. Mean time to presentation for symptomatic DVT was 19.5 days (SD +/− 21) and 25.5 days (SD +/− 27.6) for THA and NOF #. The single case of symptomatic DVT after TKA presented at 74 days post-operation.

The number of confirmed symptomatic PE were 9 for THA (9 out of 1954, 0.46%), 5 for TKA (5 out of 1870, 0.27%) TKR and 14 for NOF # (14 out of 1451, 0.96%) (Table 1).

**Table 1:** A summary of the total number (No) of operations, number of patients undergoing investigation for VTE and number of diagnostic tests positive for pulmonary embolism (PE) or deep vein thrombosis (DVT)

	No of operations	No of scans for PE	No of positive PE (%)	No of US for DVT	No of positive DVT (%)	Total no of VTE (%)
THA	1954	18	9 (0.46)	52	15 (0.77)	24 (1.23)
TKA	1870	12	5 (0.27)	86	1 (0.05)	6 (0.32)
NOF#	1451	36	14 (0.96)	67	8 (0.55)	22 (1.52)

There were 15 cases (15 out of 1954, 0.77%) of symptomatic DVT in the THA group. There was 1 case (1 out of 1870, 0.05%) of DVT in the TKA patients and 8 cases (8 out of 1451, 0.55%) of DVT in the NOF # patients (Table 1).

The overall incidence of VTE was 1.23% (24 out of 1954), 0.32% (6 out of 1870) and 1.52% (22 out of 1451) in THA, TKA and NOF # respectively (Table 1). No patients with VTE had been on chronic anticoagulant treatment prior to admission for recurrent VTE or other condition.

Within 1 year of admission, 12 patients died following THA (12 out of 1954, 0.6%), 12 after TKA (12 out of 1870, 0.6%) and 376 after NOF# (376 out of 1451, 25.9%).

Three hundred eighty-five out of 385 consecutive THA patients contacted remained within the CGH catchment area for at least 90 days post index operation. Three hundred eighty-four out of 385 consecutive TKA patients contacted remained within the CGH catchment area, with the single other patient dying within the 90 day post TKA period due to a non VTE aetiology. Three hundred fifteen of the 385 consecutive NOF# patients or carer’s contacted remained within the catchment area. There were 70 deaths, within 90 post-operative days, out of the 385 patients contacted, all dying within the catchment area.

## Discussion

The risks of VTE are increased following recent surgery and trauma [2, 3, 12–19]. Consensus statements and guidelines use data from studies showing a high frequency of clinically silent, but venogram positive, DVT with chemical thromboprophylaxis [1, 2, 20]. International comparison of guidelines for VTE prophylaxis in THA shows disagreement between the type of drugs recommended, the dose and duration of prophylaxis [1, 20, 21] perhaps due to disagreements regarding endpoints. It has been shown there is no significant difference in the diagnosis of VTE, haemorrhage and hospital readmission rates with different thromboprophylaxis policies [7]. A recent article has also highlighted the importance of surgeons being aware of possible industry-related funding as a source of potential conflicts of interests in reported VTE studies [22].

Without chemical thromboprophylaxis, historical data suggests the risks of asymptomatic DVT following THA range from 39 to 74% [23, 24], compared to 18–31%, with LMWH prophylaxis [23, 25]. A more recent Korean study demonstrated that the overall incidence of VTE following hip and knee arthroplasty was lower than Western studies, although rivaroxaban did still demonstrate a significant reduction in occurrence, whereas LMWH and aspirin had a higher rate of VTE compared to no prophylaxis [26]. Without chemical thromboprophylaxis, necropsy studies report fatal PE rates of 0.4% at 12 months after THA, and fatal PE rates of 4.3% at 12 months after NOF# [27]. Pooled data suggests fatal PE rates at approximately 6 months using thromboprophylaxis after THA and TKA of 0.43% [15]. On the other hand, another study has shown that there is no significant difference in the occurrence of VTE with or without chemical thrombopropylaxis following hip or knee arthroplasty.

After NOF#, pooled data at approximately 6 months suggests fatal PE rates of 1.9% for those not receiving prophylaxis compared to 1.0% for those receiving prophylaxis [15]. The ‘ESCORTE study’ reported a confirmed symptomatic VTE rate of 1.34% with a symptomatic PE rate of 0.25% at 6 months following NOF# [28]. The 90 day post-operative prevalence of symptomatic VTE in this study of 1.2, 0.4 and 1.5% in THA, TKA and NOF # respectively are similar to other studies using clinically symptomatic and imaging positive VTE as their endpoint [15, 16, 28–33]. The 90 day PE rates of 0.46% after THA in this study are comparable to the reported 90 day rates 0.68 and 0.6% using LMWH reported in the annual UK National Joint Registry (NJR) Report of 2010 [34] and 2007 [35] respectively. The 90 day PE rate of 0.27% after TKA in this study compares to the reported rate of 0.6% using LMWH in the annual UK NJR Report of 2007 [35], and overall 90 day hospitalization rates for VTE of 1.2% following TKA in a Danish population-based study [36]. The 1 year mortality rates of 0.6% after either THA or TKA in this study are comparable to that reported in the UK NJR of 0.6% [34] and 0.7% [35] after THA and 0.4% [34] and 0.5% [35] after TKR. A meta-analysis of 70 studies revealed pooled mortality rate of 0.38% using different thromboprophylaxis regimes [37]. The lowest rates of 0.2% were in patients receiving multimodal prophylaxis including regional anaesthesia, potent anticoagulants or aspirin, and in patients receiving warfarin combined with regional anaesthesia [37]. Another study showed the preferential use of aspirin was safe and effective for primary elective hip and knee replacement surgery, with lower rates of bleeding compared to warfarin. The VTE and mortality rates in this study are therefore comparable to reported studies [15, 16, 28–35, 37] despite using half the recommended dose of enoxaparin [6, 7] for the duration of hospital stay. The use of this lower dose has been shown to be associated with reduced mortality compared to institutions using a protocol using the recommended 40 mg dose [7].

The mean time to presentation for symptomatic PE was 22.5 days, 9 days and 22 days for THA, TKA and NOF # respectively. Mean time to presentation for symptomatic DVT was 19.5 days and 25.5 days for THA and NOF #. Other studies have also shown that most symptomatic VTE occurs after discharge from hospital [28, 29]. TKA patients appear to show a shorter risk period for VTE perhaps due to a shorter period of reduced venous blood flow postoperatively compared to after THA [38, 39]. Extended duration VTE prophylaxis has been recommended for a minimum of 4 weeks [1] based on the evidence of persistent risk of VTE beyond hospital stay for THA, TKR and NOF# for up-to 3 months [28, 29, 33]. Other guidelines recommend a shorter duration [2, 20, 33]. The benefits of post discharge thromboprophylaxis remain unproven [7, 16, 40–42]. An early analysis of the effect of the extended prophylaxis in the UK reveals that there has been no impact on the incidence of symptomatic PE and mortality [16]. There is evidence to suggest that the use of pre-operative VTE prophylaxis may be more important than extended prophylaxis, particularly in patients with multiple co-morbidities [43, 44]. We therefore began chemical prophylaxis for THA and TKA 12 h prior to surgery, which was continued until discharge [7].

There are considerable risks associated with the use of LMWH and other pharmaceutical anticoagulation agents, with not all risks being considered by guidelines [1, 3, 20]. The risk of bleeding is the major complication of anticoagulation therapy. However the criteria for defining bleeding, varies between studies, especially between orthopaedic and medical studies making firm conclusions difficult [2, 3, 45, 46]. Following prophylaxis with LMWH ‘major’ bleeds were noted in 1.2% and ‘minor’ bleeds in 1%, in a NOF # population with proven VTE rates of 1.34% [5]. A major concern is possible wound complications [47, 48] and, perhaps, increased peri-prosthetic infections, a difficult condition to manage [47, 49]. Thrombocytopenia [50] and, particularly, thrombocytopenia with thrombosis secondary to heparin can lead to increased thrombotic events, significant morbidity, lengthened hospital stay with associated complications and increased healthcare costs [51]. This formed the basis for our decision to use 20 mg enoxaparin once daily for prophylaxis.

A weakness of this study is that it analyses pooled data of all surgeons at CGH and ROPH and includes all types of THA, TKA and surgery for hip fractures. The symptomatic VTE data were collected on the assumption that all postoperative THA, TKA and NOF # patients who underwent surgery locally would present or be referred and investigated locally at CGH within the 90 day period. This is a similar assumption made in other studies where the study centre was a hospital in a town within a rural setting [17, 30]. We validated this assumption by contacting 385 consecutive patients for each of the 3 groups, revealing 100% of each group either remained within the catchment area for 90 postoperative days or died within 90 days, with all deaths occurring within the CGH catchment area. Patients who may have had fatal PEs prior to having a CTPA or V/Q scans would not be diagnosed and included in the reported VTE rates but would be included in the all-cause mortality rates. The mortality rates in our population are comparable to reported studies [15, 16, 28–35, 37].

## Conclusion

Our study uses a relatively simple method of collecting data regarding the incidence of symptomatic VTE in postoperative THA, TKA and NOF # patients. This is a method that can be utilised in other centres where PACS is available and patients are unlikely in the early postoperative period to travel to distant hospitals, therefore suited to rural settings. The relative ease of use can allow individual surgeons and units to closely monitor their VTE rates. The methodology can be utilised to also compare between centre(s) using different thromboprophylaxis regimes. The evidence for postoperative VTE rates, thromboprophylaxis and its complications are limited by the need for studies with large samples using symptomatic DVT, PE and fatal PEs as the endpoint, and enough power to detect uncommon complications [3, 4, 16, 18, 45]. We believe a co-ordinated study using this method, in geographically isolated centres, may provide the opportunity to help address some of these issues.

## Data Availability

The datasets used and/or analysed during the current study are available from the corresponding author on reasonable request.

## Abbreviations

DVT: Deep vein thrombosis
IRB: Institutional Review Board
NOF #: Neck of femur fracture
PACS: Picture Archiving Communications System
PE: Pulmonary Embolism
THA: Total Hip Arthroplasty
TKA: Total Knee Arthroplasty
VTE: Venous thromboembolism

## References

[1] No authors listed. National Institute for Health and Clinical Excellence. Venous Thromboembolism; reducing the risk of venous thromboembolism (deep vein thrombosis and pulmonary embolism) in inpatients admitted to hospital: methods, evidence and guidance. 2010 http://www.nice.org.uk/nicemedia/live/12695/47920/47920.pdf.

[2] Geerts WH, Heit JA, Clagett GP, Colwell CW, Anderson FA, Wheeler HB (2001). Prevention of venous thromboembolism. Chest.

[3] Warwick D, Dahl OE, Fisher WD (2008). Orthopaedic thromboprophylaxis: limitations of current guidelines. J Bone Joint Surg (Br).

[4] Haas SB, Barrack RL, Westrich G, Lachiewicz PF (2008). Venous thromboembolic disease after total hip and knee arthroplasty. J Bone Joint Surg Am.

[5] Burnett RS, Clohisy JC, Wright RW, McDonald DJ, Shively RA, Givens SA, Barrack RL (2007). Failure of the American College of Chest Physicians-1A protocol for lovenox in clinical outcomes for thromboembolic prophylaxis. J Arthroplast.

[6] No authors listed. British National Formulary (BNF) http://bnf.org/bnf/bnf/current/.

[7] Heidari N, Jehan S, Alazzawi S, Bynoth S, Bottle A, Loeffler M (2012). Mortality and morbidity following hip fractures related to hospital thromboprophylaxis policy. Hip Int.

[8] No authors listed. http://www.colchesterhospital.nhs.uk/corporate_information.shtml.

[9] Gudmundsen TE, Vinje B, Pedersen T (1990). Deep vein thrombosis of lower extremities. Diagnosis by real time ultrasonography. Acta Radiol.

[10] No authors listed (1990). Value of the ventilation/perfusion scan in acute pulmonary embolism. Results of the prospective investigation of pulmonary embolism diagnosis (PIOPED). The PIOPED Investigators. JAMA.

[11] Fleiss JL, Levin B, Paik MC (2003). Statistical methods for rates and proportions.

[12] Zuckerman JD (1996). Hip fracture. N Engl J Med.

[13] Kannus P, Niemi S, Parkkari J, Palvanen M, Vuori I, Järvinen M (1990). Hip fractures in Finland between 1970 and 1997 and predictions for the future. Lancet..

[14] Collins R, Scrimgeour A, Yusuf S, Peto R (1988). Reduction in fatal pulmonary embolism and venous thrombosis by perioperative administration of subcutaneous heparin. Overview of results of randomized trials in general, orthopedic and urologic surgery. N Engl J Med.

[15] Dahl OE, Caprini JA, Colwell CW, Frostick SP, Haas S, Hull RD, Laporte S, Stein PD (2005). Fatal vascular outcomes following major orthopedic surgery. Thromb Haemost.

[16] Jameson SS, Bottle A, Malviya A, Muller SD, Reed MR (2010). The impact of national guidelines for the prophylaxis of venous thromboembolism on the complications of arthroplasty of the lower limb. J Bone Joint Surg (Br).

[17] Dahl OE, Pedersen T, Kierulf P, Westvik AB, Lund P, Arnesen H, Seljeflot I, Abdelnoor M, Lyberg T (1993). Sequential intrapulmonary and systemic activation of coagulation and fibrinolysis during and after total hip replacement surgery. Thromb Res.

[18] Callaghan J, Dorr LD, Engh GA, Hanssen AD, Healy WL, Lachiewicz PF, Lonner JH, Lotke PA, Ranawat CS, Ritter MA, Salvati EA, Sculco TP, Thornhill TS (2005). Prophylaxis for thromboembolic disease: recommendations from the American College of Chest Physicians--are they appropriate for orthopaedic surgery?. J Arthroplast.

[19] Heit JA, Silverstein MD, Mohr DN, Petterson TM, O'Fallon WM, Melton LJ (2000). Risk factors for deep vein thrombosis and pulmonary embolism: a population-based case-control study. Arch Intern Med.

[20] Chari A, Khokhar A, Murray D, McNally M, Pandit H (2012). Venous thromboembolism and its prophylaxis in elective total hip arthroplasty: an international perspective. Hip Int.

[21] American Academy of Orthopedic Surgeons (2011). Preventing venous thromboembolic disease in patients undergoing elective hip and knee Arthroplasty. Evidence-based guideline and evidence report.

[22] Lee Y-K, Chung CY, Koo K-H, Lee KM, Ji H-M, Park MS (2012). Conflict of interest in the assessment of thromboprophylaxis after total joint arthroplasty: a systematic review. J Bone Joint Surg Am.

[23] Freedman KB, Brookenthal KR, Fitzgerald RH, Williams S, Lonner JH (2000). A meta-analysis of thromboembolic prophylaxis following elective total hip arthroplasty. J Bone Joint Surg Am.

[24] Coventry MB, Nolan DR, Beckenbaugh RD (1973). “delayed” prophylactic anticoagulation: a study of results and complications in 2,012 total hip arthroplasties. J Bone Joint Surg Am.

[25] Eriksson BI, Zachrisson BE, Teger-Nilsson AC, Risberg B (1988). Thrombosis prophylaxis with low molecular weight heparin in total hip replacement. Br J Surg.

[26] Yhim HY, Lee J, Lee JY, Lee JO, Bang SM (2017). Pharmacological Thromboprophylaxis and its impact on venous thromboembolism following total hip and knee arthroplasty in Korea: a nationwide population based study. PLoS One.

[27] Sheppeard H, Henson J, Ward DJ, Case CP, O’Connor BT (1981). A clinico-pathological study of fatal pulmonary embolism in a specialist orthopaedic hospital. Arch Orthop Trauma Surg.

[28] Rosencher N, Vielpeau C, Emmerich J, Fagnani F, Samama CM, ESCORTE group (2005). Venous thromboembolism and mortality after hip fracture surgery: the ESCORTE study. J Thromb Haemost.

[29] Bjornarå BT, Gudmunsen TE, Dahl OE (2006). Frequency and timing of clinical venous thromboembolism after major joint surgery. J Bone Joint Surg (Br).

[30] Dahl OE, Gudmundsen TE, Haukeland L (2000). Late occurring clinical deep vein thrombosis in joint-operated patients. Acta Orthop Scand.

[31] Fender D, Harper WM, Thompson JR, Gregg PJ (1997). Mortality and fatal pulmonary embolism after primary total hip replacement. Results from a regional hip register. J Bone Joint Surg (Br).

[32] Douketis JD, Foster GA, Crowther MA, Prins MH, Ginsberg JS (2000). Clinical risk factors and timing of recurrent venous thromboembolism during the initial 3 months of anticoagulant therapy. Arch Intern Med.

[33] White RH, Romano PS, Zhou H, Rodrigo J, Bargar W (1998). Incidence and time course of thromboembolic outcomes following total hip or knee arthroplasty. Arch Intern Med.

[34] No authors (2010). National Joint Registry 7th Annual Report, UK.

[35] No authors (2007). National Joint Registry 5th Annual Report, UK.

[36] Pedersen AB, Mehnert F, Johnsen SP, Husted S, Sorensen HT (2011). Venous thromboembolism in patients having knee replacements and receiving thromboprophylaxis: a Danish population-based follow-up study. J Bone Joint Surg Am.

[37] Poultsides LA, Gonzalez Della Valle A, Memtsoudis SG, Ma Y, Roberts T, Sharrock N, Salvati E (2012). Meta-analysis of cause of death following total joint replacement using different thromboprophylaxis regimens. J Bone Joint Surg (Br).

[38] McNally MA, Bahadur R, Cooke EA, Mollan RA (1997). Venous haemodynamics in both legs after total knee replacement. J Bone Joint Surg (Br).

[39] McNally MA, Cooke EA, Mollan RA (1997). The effect of active movement of the foot on venous blood flow after total hip replacement. J Bone Joint Surg Am.

[40] Bergqvist D, Jönsson B (2000). Cost-effectiveness of prolonged out-of-hospital prophylaxis with low-molecular-weight heparin following total hip replacement. Haemostasis..

[41] Skedgel C, Goeree R, Pleasance S, Thompson K, O'brien B, Anderson D (2007). The cost-effectiveness of extended-duration antithrombotic prophylaxis after total hip arthroplasty. J Bone Joint Surg Am.

[42] Eikelboom JW, Quinlan DJ, Douketis JD (2001). Extended-duration prophylaxis against venous thromboembolism after total hip or knee replacement: a meta-analysis of the randomised trials. Lancet..

[43] Lie SA, Engesaeter LB, Havelin LI, Furnes O, Vollset SE (2002). Early postoperative mortality after 67,548 total hip replacements: causes of death and thromboprophylaxis in 68 hospitals in Norway from 1987 to 1999. Acta Orthop Scand.

[44] Borgen PO, Dahl OE, Reikeras O (2010). Preoperative versus postoperative initiation of dalteparin thromboprophylaxis in THA. Hip Int.

[45] Neviaser AS, Chang C, Lyman S, Della Valle AG, Haas SB (2010). High incidence of complications from enoxaparin treatment after arthroplasty. Clin Orthop Relat Res.

[46] Levine MN, Raskob G, Beyth RJ, Kearon C, Schulman S (2004). Hemorrhagic complications of anticoagulant treatment: the seventh ACCP conference on antithrombotic and thrombolytic therapy. Chest..

[47] Aquilina AL, Brunton LR, Whitehouse MR, Sullivan N, Blom AW (2012). Direct thrombin inhibitor (DTI) vs. aspirin in primary total hip and knee replacement using wound ooze as the primary outcome measure. A prospective cohort study. Hip Int.

[48] Patel VP, Walsh M, Sehgal B, Preston C, DeWal H, Di Cesare PE (2007). Factors associated with prolonged wound drainage after primary total hip and knee Arthroplasty. J Bone Joint Surg Am.

[49] Parvizi J, Ghanem E, Joshi A, Sharkey PF, Hozack WJ, Rothman RH (2007). Does “excessive” anticoagulation predispose to periprosthetic infection?. J Arthroplast.

[50] Kejariwal D (2006). Heparin-induced thrombocytopenia: a complication of thromboprophylaxis. J Bone Joint Surg (Br).

[51] Baroletti S, Piovella C, Fanikos J, Labreche M, Lin J, Goldhaber SZ (2008). Heparin-induced thrombocytopenia (HIT): clinical and economic outcomes. Thromb Haemost.

